# Barriers and facilitators for safe sex behaviors in students from universidad de Santiago de Chile (USACH) through the COM-B model

**DOI:** 10.1186/s12889-023-15489-y

**Published:** 2023-04-11

**Authors:** Manuel Armayones Ruiz, Eduardo Leiva Pinto, Oriana Figueroa, Noemí Robles, Denise Laroze Prehn, Francisco Villarroel Riquelme, Giuliano Duarte Anselmi

**Affiliations:** 1 eHealth Center, Open University of Catalonia, Barcelona, Spain; 2 Faculty of Human Sciences, Universidad Bernardo O’Higgins (UBO), Santiago, Chile; 3 Social Complexity Sciences, Center for Research in Social Complexity, Universidad del Desarrollo, Santiago, Chile; 4 Department of Management, Faculty of Economics and Management, Center for Experimental Social Sciences, Universidad de Santiago de Chile (USACH), Santiago, Chile; 5 Faculty of Medical Sciences, School of Obstetrics and Childcare, Universidad de Santiago de Chile, Santiago, Chile

**Keywords:** Youth sexuality, Safe sex, Barriers and facilitators, STI/HIV prevention, TDF, COM-B

## Abstract

**Background:**

Unsafe sex is one of the main morbidity and mortality risk factors associated with sexually transmitted infections (STIs) in young people. Behavioral change interventions for promoting safe sex have lacked specificity and theoretical elements about behavior in their designs, which may have affected the outcomes for HIV/AIDS and STI prevention, as well as for safe sex promotion. This study offers an analysis of the barriers and facilitators that, according to the university students who participated in the focus groups, impede or promote the success of interventions promoting healthy sexuality from the perspective of the actions stakeholders should undertake. In turn, this study proposes intervention hypotheses based on the Behavior Change Wheel which appears as a useful strategy for the design of intervention campaigns.

**Methods:**

Two focus groups were organized with students from Universidad de Santiago de Chile (USACH). The focus groups gathered information about the perceptions of students about sex education and health, risk behaviors in youth sexuality, and rating of HIV/AIDS and STI prevention campaigns. In the focus groups, participants were offered the possibility of presenting solutions for the main problems and limitations detected. After identifying the emerging categories related to each dimension, a COM-B analysis was performed, identifying both the barriers and facilitators of safe sex behaviors that may help orient future interventions.

**Results:**

Two focus groups were organized, which comprised 20 participants with different sexual orientations. After transcription of the dialogues, a qualitative analysis was performed based on three axes: perception about sex education, risk behaviors, and evaluation of HIV/AIDS and STI prevention campaigns. These axes were classified into two groups: barriers or facilitators for safe and healthy sexuality. Finally, based on the Behavior Change Wheel and specifically on its ‘intervention functions’, the barriers and facilitators were integrated into a series of actions to be taken by those responsible for promotion campaigns at Universidad de Santiago. The most prevalent intervention functions are: education (to increase the understanding and self-regulation of the behavior); persuasion (to influence emotional aspects to promote changes) and training (to facilitate the acquisition of skills). These functions indicate that specific actions are necessary for these dimensions to increase the success of promotional campaigns for healthy and safe sexuality.

**Conclusions:**

The content analysis of the focus groups was based on the intervention functions of the Behavior Change Wheel. Specifically, the identification by students of barriers and facilitators for the design of strategies for promoting healthy sexuality is a useful tool, which when complemented with other analyses, may contribute improving the design and implementation of healthy sexuality campaigns among university students.

**Supplementary Information:**

The online version contains supplementary material available at 10.1186/s12889-023-15489-y.

## Introduction

Unsafe sex is one of the main morbidity and mortality risks in young people [1] as it is associated with sexually transmitted infections (STIs), including infection by the human immunodeficiency virus (HIV). The main prevention mechanism is behavioral, through safer sexual practices like the use of condoms. Therefore, understanding this behavior and the context where it takes place is essential for designing theory- and evidence-based behavioral interventions [2] And although there is evidence on combined interventions that promote HIV/STI interventions (biomedical, behavioral and structural) [3] [4], our work will center on behavioral interventions.

These behavioral change interventions require that young people take specific actions to achieve this change, for example, using condoms, gathering information about health consequences, requesting the support of their partners and peer groups, and even redesigning the environment to facilitate access to condoms and HIV testing [5]. These actions are different for each individual, community or context, and thus it is necessary to know the barriers that impede or encourage these specific actions to perform interventions that enhance skill acquisition, increase the motivation to carry out these actions, and make sure that both the physical and social environment are open to them [6].

The prevalence of HIV in the world is heterogeneous; 60% of cases are concentrated in Sub-Saharan Africa, with 4.000 new infections each day. Of these, 51% affect women and 31% young people aged 15 to 24. Currently, 37.7 million people are living with HIV in the world. In 2020, there were 1.5 million new cases of HIV in the world and 680,000 deaths associated with AIDS [7].

UNAIDS’ 2020 goals for reducing the number of new cases and deaths associated with HIV were not met [8]. This is partly related to the coronavirus pandemic (COVID-19). In Latin America, the percentage change of new infections from 2010 to 2020 was 0%, i.e., there was no reduction in new infections; they have remained the same. However, there was a 63% increase in Chile during the same period [7].

According to the 2021 UNAIDS report, Chile has a 0.6 prevalence in the adult population aged 15 to 49, i.e., 6 in 1,000 people live with HIV. The risk of becoming infected with an STI or HIV is not always considered by young people, and their knowledge does not always lead to preventive practices. According to UNAIDS, at the regional level, Chile presents the highest incidence of STI/HIV in Latin America, and young people present a particularly high risk of infection. At the national level, the age brackets with the highest rate of HIV, gonorrhea, hepatitis B, and syphilis are the 5-year brackets of 20–24 and 25–29, where most of the university population is categorized [9].

Regarding prevention, the WHO recommends a comprehensive set of health services for the prevention of STI/HIV, among which are voluntary male circumcision, pre-exposure prophylaxis (PrEP), post-exposure prophylaxis (PEP), and the use of condoms as the main method for this, reducing by 94% the possibility of transmission [10]. However, according to the last data reported in the 2016–2017 National Health Survey, only 1 of 5 young people would use condoms in Chile [11]. Joint and multidisciplinary efforts are needed in terms of prevention to tackle the obstacles faced by university students in the acquisition of behaviors for safe sex.

Among the preventive strategies generated by different organizations and/or governments, individual, group, and community interventions are identified. Regardless of the level of intervention, all share a common goal: to modify the behavior of individuals, identifying both the barriers to achieving health goals, as well as the facilitators based on the positive outcomes of the campaigns.

### Aims and objectives

This study has two objectives: first, to analyze the barriers and facilitators that, according to students, impede or favor the success of interventions for the promotion of healthy sexuality; and second, to propose a series of actions based on the analysis of barriers through the Behavior Change Wheel model [12]. Through the latter, we aim to offer structured intervention proposals for health promotion directly based on feedback from young people, who are their potential receptors.

## Methods

### Behavior change wheel

**Fig. 1:**
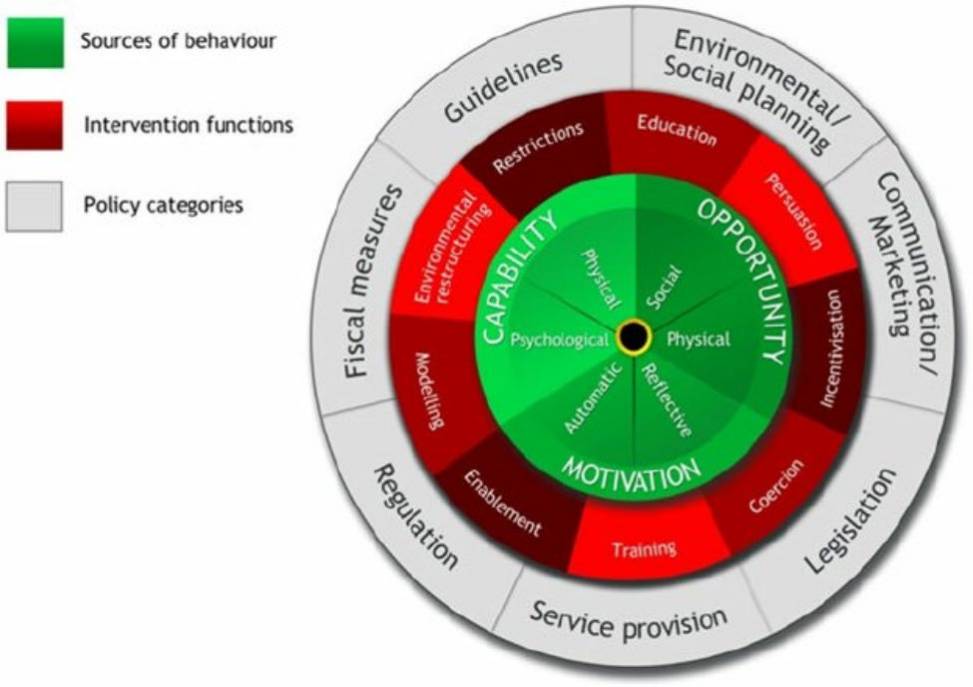
Behavior Change Wheel Source: Michie, S., Atkins, L., & West, R. (2014).

The Behavior Change Wheel (BCW, hereinafter) defines behavior as an interaction between three necessary conditions: (1) Capacity to perform the behavior; (2) Physical or social opportunity to perform the behavior, and; (3) Motivation to perform the behavior. The three variables form the COM-B model (Fig. 1).

Around its nucleus, there is a second layer that comprises 9 intervention functions. Intervention functions are broad categories of means through which an intervention may change behavior. Intervention functions are the following: education, persuasion, incentivization, coercion, training, enablement, modeling, environmental restructuring, and restrictions.

In turn, these functions are linked to the dimensions of COM-B, generating more or less psychological and physical capacities, external or automatic motivation, or physical and/or social opportunity, as seen in Table 1.

**Table 1:** Relationship between intervention functions of the BCW model and the dimensions of COM-B (in bold)

Intervention	Characteristics
Education	It can increase both the **psychological capacity** (for understanding, self-regulation, memory, and attention) and the **physical capacity** to perform a behavior. It can also promote reflexive motivation.
Training	It can increase **psychological capacity**, as well as improve «**physical opportunity**» and **automatic motivation** through the creation of habits.
Restriction	It can modify the **physical and social opportunities** of an individual or group.
Environmental restructuring	It can modify the **physical and social capacities** of individuals, as well as their **automatic motivation.**
Modeling	Useful for increasing both **reflexive motivation** and those elements related to **social opportunity** (being influenced by the social norms of the group).
Enablement	It can increase the **physical and psychological capacity**, as well as the **physical opportunities** and the **automatic motivation**, of an individual, generating «facilitators» in the context for the behavior to be performed.
Coercion	It reduces the physical **capacity** to act due to the threat this poses to the individual or group.
Incentivization	It increases both physical capacity and **extrinsic motivation** through the expectation of some physical or social reward. It can also increase **intrinsic motivation** (habit creation).
Persuasion	It can increase the **extrinsic motivation** of the individual by appealing to their emotions.

Source: Michie, S., Atkins, L., & West, R. (2014).

The last layer of the wheel, the most external, comprises seven policy categories (environmental/social planning, communication, legislation, service provision, regulation, fiscal measures, and guidelines) that can be used to support the realization of the intervention functions, but that we will not address in this study. The interaction of these layers may provoke a behavior change, and therefore the behavioral wheel model can be employed as a tool for both designing and evaluating interventions.

#### Design

A descriptive qualitative design was applied, based on collecting information from the target population. Then, focus groups were organized and the information gathered from them was analyzed using content analysis. Finally, the content was classified into three categories or thematic axes: (a) Perceptions about sex education and sexual health; (b) risk behaviors in sexuality; (c) evaluation of STI/HIV prevention campaigns. The script of the focus groups is presented in Appendix 1.

#### Participants

The target population in our study were students from Universidad de Santiago de Chile (USACH) who attended the programs of Obstetrics, International Studies, English Teaching, Mathematical Engineering, Chemical Engineering and Industrial Design. A group of 40 students were invited to participate in the focus groups. Student representation organizations from USACH and representatives from Student Gender and Sexuality Society (VGS hereinafter) from the same university collaborated with the recruitment process. VGS representatives met the research team several times to learn the fundamentals and scope of the study in detail. These actions enabled the formation of a good rapport and the incorporation of the groups into the study as a specific advisory board, participating in future co-created interventions.

This study used a purposive sample. VGS reached out to students via social networks and e-mails. In a meeting, VGS informed the students who responded to the terms of the research. The research team offered VGS guidelines for participant selection, seeking the highest levels of diversity and complementariness in the approach to the main theme axes and information given. To establish the sample, the saturation principle was employed following Mayan [13]. In this way, saturation was conceived as an analytical instance of an intersubjective approach between the research team and the population participating in the study. Saturation was understood as well as an appraisal of the experience that was aimed at elucidating most aspects of the studied matter from varied significant perspectives. Saturation was achieved from an analytical and procedural perspective, as well as the density and veracity of the information. [14] [15].

Participants did not know the researchers nor the research lines of the study.

#### Procedure

Two focus groups were organized, both were conducted on the premises of Universidad de Santiago de Chile (USACH) [16]. The groups were led by researchers from USACH, Giuliano Duarte Anselmi, midwife and public health professional, and Eduardo Leiva Pinto, medical anthropologist and expert in qualitative methodologies, while members of the Ethics Committee of USACH participated as observers. Participants, students of the USACH, signed an informed consent prior to the session, and sociodemographic data were collected for sample characterization. The procedure was approved by the Ethics Committee of USACH.

The focus groups were conducted considering the following thematic axes: (a) Perceptions about sex education and sexual health; (b) risk behaviors in sexuality; (c) evaluation of STI/HIV prevention campaigns. The script was piloted with students from USACH and VGS members.

### Data collection

The sessions were recorded in audio and then transcribed. Transcripts were presented to the VGS members. Subsequently, two researchers (OF, FV) performed an iterative thematic analysis based on the two initial categories:

- **Behavioural Barriers** for young people to have safe sex.
  In this study, barriers are understood as those aspects related to the perceptions about sex education and sexual health, risk behaviors in sexuality and evaluations of STI/HIV prevention campaigns that according to participants made healthy sex education and sexuality difficult.
- **Behavioural Facilitators** for young people to have safe sex.
  In this study, facilitators are understood as those aspects related to the perceptions about sex education and sexual health, risk behaviors in sexuality and evaluations of STI/HIV prevention campaigns that according to participants would facilitate healthy sex education and sexuality.

Two researchers (OF and FV) analyzed the transcribed qualitative data collected in the two focus groups, and made a deductive classification for “barriers” and “facilitators”. The work of both was blinded and independent. Both classifications were then put together and if both matched the classification, they were included as such.

Then, a third researcher (MA) identified the inconsistencies between the classifications of the two researchers who coded the data and solved them in a meeting with them, integrating the work of both OF and FV into a single document that was validated through a second meeting with them.

The analysis was conducted manually by the researchers, without support from any specialized software.

Once all interventions were classified into one of the two categories, a specific table was created for each, namely for barriers (Table 2) and facilitators (Table 3). It should be noted that the “facilitators” table contains actions that, according to individuals, contributed to neutralize some of the barriers detected, whether these are coincident or not with the barriers in Table 1.

**Table 2:** Barriers perceived in terms of the systematization axes

Systematization axis	Code	Perceived barriers
**Perceptions about sex education and sexual health**	B1	There is a bad sex education: superficial and associated with punishment
	B2	Limited training in sexual and reproductive health among teachers
	B3	Sex education-centered on biological aspects.
	B4	Health professionals spread “stigmas” and prejudice.
	B5	Campaigns do not include “forgotten” STIs.
	B6	Campaigns with very gloomy content.
	B7	Ignorance about the correct way to use condoms.
	B8	Male-chauvinist attitudes towards the non-use of condom.
	B9	Prejudice about the use of vaginal condoms.
	B10	Lack of medical assistance culture in sexuality.
	B11	View of condoms associated with contraception.
	B12	Excess of information on the Internet makes it difficult to discern quality information.
	B13	View of sexuality as a taboo.
	B14	Communication problems related to sexuality in families.
	B15	Absence of campaigns that are practical and respond to real needs.
	B16	Generalized misinformation of the population (adolescents and teachers)
	B17	Insufficient conditions for preventive screening at the university.
	B18	Barriers in health care centers for obtaining condoms.
	B19	Lack of training from staff to deal with sexual dissidents (intersex, queer, trans)
	B20	Prejudice from health care staff at the moment of conducting medical screenings.
	B21	Engineering students do not talk about sexuality and prevention.
	B22	Lack of adequate condom dispensers (type of coin accepted)
	B23	Condom dispensers are difficult to access.
	B24	Ignorance about vaginal condoms.
	B25	Ignorance about the existence of condom dispensers by the community.
**Risk behaviors in sexuality**	B26	People who consume alcohol don’t use condom during sexual relations.
**Evaluation of STI/HIV prevention campaigns**	B27	Campaigns centered on fear of HIV.
	B28	Teaching the risks of HIV by “risk groups” is counterproductive and stigmatizing.
	B29	Prejudicial campaigns that end up spreading misinformation.
	B30	Taboos among professionals to explain how to use condoms.
	B31	Ignorance of the price of and access to condoms.
	B32	Information from media is loaded with prejudice and misinformation.
	B33	Campaigns too centered on the consequences of the disease.
	B34	Campaigns only aimed at heterosexual audiences.
	B35	Campaigns focus on sexual abstinence or exclusive partner.
	B36	Taboos in teaching how to use penis or vaginal condoms.
	B37	Prejudice of young people about the quality of condoms offered by the public health system.
	B38	Ignorance about what is a risk behavior.

**Table 3:** Facilitators in terms of systematization axes

Systematization axis	Code	Facilitators
Perceptions about sex education and sexual health	F1	Implementing sexual health campaigns at all levels.
	F2	Indicate appointments with a midwife as the best informative opportunity.
	F3	Have a smartphone app to access reliable information.
	F4	Promote the use of condoms to prevent pregnancy.
	F5	Organize STI prevention activities for all groups and ages.
	F6	Indicate midwives as sources of information.
	F7	Normalize asking for STI screening without prejudice, offense, or taboos, both for oneself and one’s partner.
	F8	Create a counseling service pre- and post-HIV-test.
	F9	Create safe points for obtaining condoms at events for all genders, with instructions for use.
	F10	Offer strategies to talk with friends about the importance of screening.
	F11	Increase women’s empowerment to talk about the use of condoms.
	F12	Show how to use penis or vaginal condoms.
	F13	Use illustrated instructions next to dispensers to show the correct way to use condoms
	F14	To increase training regarding the female condom
Risk behaviors in sexuality	F15	Increase the affective responsibility for sex among young people.
	F16	Encourage being assertive in requesting potential partners’ negative tests for STIs.
Evaluation of STI/HIV prevention campaigns	F17	That campaigns offer a comprehensive view of sexuality beyond penetration: emotional and responsible.
	F18	That the role of pleasure in sexuality is underscored as a fundamental part of people’s lives.
	F19	That campaigns are simple, informative, and free of prejudice.
	F20	That campaigns show the flexibility of condoms.
	F21	Capacity to verbalize HIV + status.
	F22	That speaking about sexual rights and reproductive health in campaigns is easy.
	F23	That campaigns with updated content are offered.
	F24	That campaigns acknowledge the mismatch between sexual practices and sexual orientation.
	F25	That campaigns break apart from the heteronormative logic of sex.
	F26	That campaigns offer information about risk behaviors even when in a monogamous relationship.
	F27	That campaigns do not fall into prejudice when informing about the risk of some sexual practices.
	F28	That campaigns are addressed based on social responsibility.
	F29	That campaigns have a design that promotes adherence (for example, gamification elements)
	F30	That spaces for accessing information are people-friendly.
	F31	That campaigns offer concrete information and responses for each person.
	F32	That campaigns are not only centered on HIV (also addressing other STIs).
	F33	That campaigns focus on skills for self-care.
	F34	That campaigns offer models to identify with.

Actions were classified by researchers NR and MA, into the three dimensions based on the description of the dimensions of the COM-B model included in the Behaviour Change Wheel: capacity, opportunity and motivation.

## Results

Out of the 40 students reached, 20 participated in the study. Students who did not show up (8) and those who refused to participate (12) were removed from the sample.

Participants were aged 18 to 25 years. Most of the sample consisted of women (n=14), followed by men (n=5), and 1 participant who reported having a non-conformant gender (n=1). Regarding sexual preferences, participants reported being heterosexual (12), lesbian (4), homosexual (3), and bisexual (1). 

The main results of the focus groups are presented following the COREQ criteria [17]. We include an overview of the full COREQ criteria in Appendix 2.

Once all interventions were classified into one of the two categories (barriers and facilitators), a specific table was created for each, namely for barriers (Table 2) and facilitators (Table 3) grouped by the three systematization axis: perceptions about sex education and sexual health, risk behaviors in sexually and evaluation of STI/HIV prevention campaigns.

In order to offer some concrete recommendations about actions to be taken in future sexual health promotion campaigns, a specific table was created (Table 4). **In this table** all the facilitator actions from Table 3, were integrated; in addition to Table 2 barriers, that were rewritten as positive and specific actions to be potentially taken in Table 4, and linked by the intervention functions of Behaviour Change Wheel Framework. (op. cit.)

**Table 4:** Integration of barriers and facilitators classified based on COM-B dimensions,

Integration of barriers and facilitators classified based on COM-B dimensions, indicating the intervention function recommended.			
COM-B dimension	Code	Required action B: Behavioral reformulation of T2:Barriers F: Behavioral formulation of T3: Facilitator	INTERVENTION FUNCTION ASSOCIATED
Psychological capability	B1	Improve sex education without making it superficial or associated with punishment	Education
	B2	Improve the training of sex and reproductive health education teachers	Education
	B3	Not centering sex education only on biological aspects	Education
	B8	Diminishing male chauvinist attitudes toward the use of condoms	Persuasion
	B9	Design campaigns without prejudice against the female condom	Persuasion
	B12	Offer information that allows for distinguishing quality information on the Internet	Education
	B15	Increase clear information among adolescents and young people	Education
	B19	Train the staff to address people who are sexual dissidents (intersex, queer, trans)	Education
	B21	Design specific training for engineering students	Education
	B24	Improve knowledge about vaginal condoms.	Education
	B25	Improve the knowledge of the community about the existence of condom dispensers	Education
	B26	Teach about the relationship between alcohol consumption and condoms	Education
	B31	Training in price and access to condoms	Education
	B38	Training in what is a risk behavior	Education
	F4	Promote the use of condoms to prevent pregnancy	Education
	F10	Offer strategies for talking with friends about the importance of screening	Training
	F14	Increase training in the use of vaginal condoms	Education
	F16	Be assertive to request potential partners’ negative STI test results	Training
	F21	Capability to verbalize an HIV + status	Training
	F23	Offer campaigns with updated content	Education
	F26	Offer campaigns with information about risk behaviors even if in a monogamous relationship	Education
	F31	Campaigns that offer concrete information and responses for each person.	Education
	F33	Campaigns that focus on skills for self-care.	Training
Physical capability	B7	Create workshops about the correct use of condoms	Training
	B16	Facilitate campaigns and practical workshops oriented to real needs	Training
	F9	Create safe points for obtaining condoms at events for both genders, with instructions for use.	Environmental restructuring
Social opportunity	B13	Offer models that do not depict sexuality as a taboo	Modeling
	B14	Create dialogue spaces for sexuality in the family	Enablement
	B32	Training in the fact that information from the media is loaded with prejudice and misinformation.	Education
	F7	Generate systems to request STI screening without prejudice, offense, or taboos, both for oneself and for one’s partner.	Enablement
	F8	Create a counseling service pre- and post-HIV-test.	Enablement
	F11	Increase women’s empowerment to talk about the use of condoms	Persuasion
	F22	Enable speaking about sexual rights and reproductive health in campaigns.	Education
	F24	Campaigns that acknowledge the mismatch between sexual practices and sexual orientation	Persuasion
	F25	Campaigns that break away from the heteronormative logics of sex.	Persuasion
	F28	Campaigns that address social responsibility.	Persuasion
	F29	That campaigns have a design that promotes adherence (for example, gamification elements)	Persuasion
	F34	Campaigns that offer models to identify with.	Modeling
Physical opportunity	B17	Improve conditions for preventive screening at the University	Environmental restructuring
	B18	Reduce barriers in health care centers for obtaining condoms.	Environmental restructuring
	B22	Improve access to condom dispensers (type of coin accepted)	Environmental restructuring
	B23	Improve access to condom dispensers (location)	Environmental restructuring
	B36	Eliminate the taboo of teaching how to use male or female condoms.	Training
	F3	Create smartphone apps to access reliable information	Enablement
	F12	Show how to use penis or vaginal condoms.	Training
	F13	Use computer graphics next to dispensers to show the correct way to use condoms	Training
	F20	Show flexibility of condoms in campaigns.	Modeling
	F30	Make spaces for accessing information people-friendly.	Environmental restructuring
Reflexive motivation	B4	Train professionals so they do not spread “stigmas” and prejudice.	Persuasion
	B5	Include “forgotten” STIs in trainings.	Education
	B10	Promote a culture of medical care in sexuality	Incentivization
	B20	Train professionals so they don’t show prejudice when conducting medical screenings.	Persuasion
	B27	Create awareness that campaigns are too centered on fear of HIV.	Persuasion
	B28	Avoid teaching the risks of HIV by “risk groups”	Education
	B29	Eliminate prejudice from campaigns to avoid misinformation	Persuasion
	B30	Neutralize taboos about how to use condoms among professionals	Persuasion
	B33	Promote campaigns that are not only centered on the consequences of the disease.	Education
	B34	Promote campaigns not only aimed at heterosexual audiences	Education
	B35	Eliminate campaigns focused on sexual abstinence or exclusive partners	Education
	B37	Intervene to eliminate the prejudice of young people about the quality of condoms offered by the public health system.	Persuasion
	F1	Implementing sexual health campaigns at all levels.	Education
	F2	Indicate appointments with a midwife as the best informative opportunity.	Persuasion
	F5	Organize STI prevention activities for all groups and age brackets.	Education
	F15	Increase the affective responsibility for sex among young people.	Education
	F17	Offer campaigns with a comprehensive view of sexuality beyond penetration: emotional and responsible.	Education
	F18	Highlight the role of pleasure in sexuality as a fundamental part of people’s lives.	Education
	F19	Make campaigns simple, informative and free of prejudice.	Education
	F32	Center campaigns not only on HIV (also addressing other STIs)	Education
Automatic motivation	B11	Break away from the association of condom and contraception	Persuasion

The selection of the different «function interventions» was based on the link matrix between COM-B and intervention functions created by Michie, Atkins and West [18]. The researchers OF, FV and MA discussed the classification of the different facilitators and barriers to the COM-B categories (capability, opportunity, or motivation) so those responsible for the health promotion campaigns could perform the behaviors.

Actions were classified by researchers NR and MA, into the three dimensions based on the description of the dimensions of the COM-B model included in the Behaviour Change Wheel: capacity, opportunity, and motivation.

**Fig. 2:**
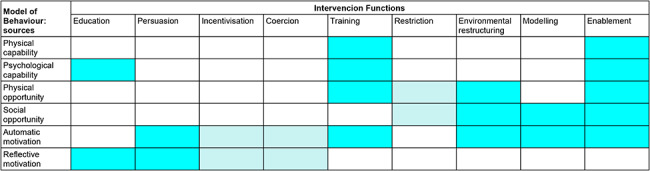
Relationship between model of behaviour sources and Intervention Functions Source: Authors' elaboration based on Michie, S., Atkins, L., & West, R. (2014). Note: Color intensity represents better matching of the intervention function to the behavior source.

Grouping the results by intervention functions, following the recommendations of Michie et al. (op. cit.) and represented in Fig. 2; Table 5 shows the distribution, in increasing order, of actions and interventions that young people believe should be maintained (facilitators) or should be improved (barriers transformed into actions to be taken) in the intervention campaigns for the promotion of healthy sexuality. As Table 5 shows, Education is by far the most commonly used Intervention function (46.9%), followed by Persuasion (19.64%) and Training (13.63%). Other interventions such as Incetivation, Coercion and Restriction were never used.

**Table 5:** Intervention functions

Intervention function	No. of actions	Percentage
Education	31	46.9%
Persuasion	13	19.64%
Training	9	13.63%
Environmental restructuring	6	9.09%
Enablement	4	6.06%
Modeling	3	4,34%
Incentivisation	0	-
Coercion	0	-
Restriction	0	-

Of the 66 actions presented, which should be incorporated in the interventions for promoting healthy and safe sex among young people according to students, 31 (46.9%) were interventions related to the ‘Education’ intervention function, which according to the Behavior Change Wheel (op. cit.), are related to the increase in knowledge and understanding about a specific topic. This result is consistent with this type of study and the structure of its focus groups, which inquired directly about the evaluation of campaigns, perceptions about sexuality, and risky sex behaviors.

Regarding the intervention function of education, it should be noted that participants believe that campaigns should be oriented differently from what has been done in the past and that these should be designed based on quality information, tailored to different groups and their needs. Campaigns should also be delivered by technically trained professionals who hold no prejudices. In addition, campaigns should not be centered only on the biological aspects of sexuality but also address the different expressions of the same, going beyond the description of the possible risks of sex, as this often makes campaigns center on HIV instead of other STIs according to the participants. Furthermore, it is essential to emphasize that sexual relations are a source of pleasure and offer practical rather than only theoretical information.

The second intervention function emerges from the analysis of ‘persuasion’ (19.64%). In the Behavior Change Wheel, persuasion is used as a communicative strategy that induces positive or negative emotions, or even action. In this study, persuasion is related to the need to sometimes influence the trainers themselves so they do not transmit prejudices—for example, about the use of the female condom, or some male-chauvinist attitudes towards the use of condoms during sexual relations—to campaigns. It is also considered vital to influence women’s empowerment in terms of prophylactic measures such as the female condom or even to convince the community that there is no relationship between sexual practices and sexual orientation. Additionally, campaigns should break away from the heteronormative approach to sex and address the topic from a social responsibility perspective. Finally, campaigns should also have elements that promote adherence, for example, gamification, i.e., they should generate positive experiences among people who follow campaigns to promote future adherence.

The intervention function of ‘training’ is the performance of actions aimed at the acquisition of specific competencies according to the BCW.

In the results, this intervention function accounts for 13.63% of the proposed actions (13 actions). This intervention function applies to the proposal of participants for campaigns to have a marked practical nature in terms of offering young people strategies for talking with their friends about the importance of preventive screenings, the capability of verbalizing an HIV + status, and the assertiveness to ask for potential sexual partners negative test results. Participants also indicate the need for enabling the learning of competencies for their own care, and that workshops should be practical in terms of demonstration of the use of female and male condoms, and the creation of materials that show in the most practical way how to use protective devices against STIs.

The intervention function of ‘environmental restructuring’ (with 6 possible actions) accounts for 9.09% of proposals. In this sense, this result is interesting because it reinforces the idea of identifying concrete actions to take in the physical space.

The actions related to ‘environmental restructuring’ refer to aspects such as enabling physical points to deliver condoms safely, reducing barriers, and improving the access and location of condom dispensers, as well as enhancing the conditions for preventive screenings at the university both in terms of location and friendliness of the staff.

Other intervention functions like ‘enablement’ represent 6% of all actions indicated by students (concretely 4). It is noteworthy that ‘enablement’ in the BCW comprises the creation of services and devices that “enable” access to services. Some students indicate that there should be spaces for dialogue with the family, for pre- and post-STI tests follow-ups, and for the generation of specific mobile apps that students can use.

## Discussion

The fact that the analysis of barriers and facilitators identified by the students and subsequently processed through the Behavior Change Wheel incorporate such basic intervention functions as education (46.9%), persuasion (19.64%), and training (13.63%) reinforces the idea that students perceive sex education, the addressing of sex risks and especially the campaigns launched as having much space for improvement in terms of both their content and the way it is transmitted. In fact, students emphasize the need for going beyond knowledge, focusing on avoiding stereotypes and giving interventions a more practical approach. In other words, not centering “what” is explained so much as “how”.

Discussing our findings we found coincidences about what function intervention can address the need for sexual health promotion actions in college students in the work of Cassidy et al. [19], who identify the same functions interventions that our study informs (education, environmental restructuring, enablement, modeling, persuasion,) in a study in Canada.

Relating to education and environmental restructuring such function interventions to overcome barriers, we coincide with Bersamin et al. [20] that found that the combination between education (through a specific course) and the opportunity to attend university sexual health services highlight universities as uniquely position to reduce perceived barriers between studies.

Related to sexual health promotion, our study offers a new participative approach, based on BCW, that allows professionals and university actors to not only identify barriers to healthy sexuality, but also to detect opportunities for improvement for the entire community, making young students the main source of knowledge and specific ideas to overcome those barriers.

In the same way, going beyond the classical approach of providing information to students, they themselves identify specific ‘functions interventions’, such as training and persuasion, which offer us new ways of designing a life of healthy sexual promotion.

Chile has the highest incidence of STIs/HIV in Latin America. This is particularly worrying in young people. Interventions in sexual health have failed to address the experiential aspects of youth sexuality, valuing ideal and stereotyped behavior models, discarding first-person narratives and their complexity. Therefore, it is imperative to innovate in the design and implementation of STI/HIV prevention strategies, formulating interventions based on a multidisciplinary, integrative, and situated design that values the experience of the target populations, the reported evidence, and theory.

Our work has some limitations. The first one is related to the fact that the leaders of the focus groups were members of the research team.

This could introduce some bias in the response, which could have been avoided by having external researchers. The second limitation is that the dynamic of the focus group was based on previous categories from the experience of researchers, but not directly on the dimensions of the «sources of behavior» of the BCW (capacity, motivation and opportunity) [12].

Despite these limitations, this study is the first to be carried out in Latin America, with a representative sample of university students and where a topic has been addressed in a replicable framework and from an interdisciplinary perspective. In that sense, this research sought to capture the subjectivity of the target population of the prevention campaigns to give a voice to the people who should benefit from this type of health initiative.

## Conclusion

Through this study, we have confirmed that the COM-B methodology like a component of the whole Behaviour Change Wheel, allows for obtaining a series of concrete action proposals to implement at a first decision-making level. We used the BCW to identify which functions interventions can better address the barriers identified by students in sexual health promotion campaigns.

The strengh of our study is not only related with the specific result but with the use of a framework such Behavioural Change Wheel. Authors like Cassidy [19] used before the BCW to improve sexual health service use among university undergraduate students and in our study we confirm that BCW can be a useful tool to asses, monitor and design new intervention. And in addition, to find a new way to identify patterns of lacks, both in capacity, motivation and opportunity, and explore how these needs are adressing with different interventions.

Additionally, we consider that BCW offers both researchers and professional a set of tools, that allow to co-design interventions combining not only barriers detections but identifying facilitators from themselves and not imposed by expert criteria, improving the meaningful involvement of stakeholders in creating new knowledge and mobilising and transferring existing knowledge to support long-term solutions [21].

However, and in future work, our approach should be complemented with other sources of information, both in terms of the APEASE criteria from the Behavioural Change Wheel framework: (affordability, practicability, effectiveness/cost-effectiveness, acceptability, safety, and equity) [18] related with each intervention function to explore its appropriateness for the sexual health promotion campaigns. Additionally, further research can be useful to compare barriers and facilitators, and related functions intervention with the view of other community stakeholders such as doctors, nurses, academic staff, etc.

## Electronic supplementary material

Below is the link to the electronic supplementary material.

Supplementary Material 1

Supplementary Material 2

## Data Availability

The datasets generated and/or analysed during the current study are publicly available (in Spanish) here. https://drive.google.com/drive/folders/1fM0J-TaXMyl9678kAODgXTCw54CZzrxs?usp=sharingdue. Futher information are available by the corresponding author on reasonable request.
