# Supplementary material for: Barriers and facilitators for safe sex behaviors in students from universidad de Santiago de Chile (USACH) through the COM-B model
Source: BMC Public Health. 2023 Apr 11;23:677. doi: 10.1186/s12889-023-15489-y (PMC10088188; doi:10.1186/s12889-023-15489-y)
Supplement: Supplementary file 1 — Supplementary Material 1 [file 12889_2023_15489_MOESM1_ESM.pdf]

‘Efecto de las intervenciones digitales para la prevención de infecciones de transmisión sexual/VIH’
DICYT 2019 – Universidad Santiago de Chile, USACH

Grupo Focal N°:

Fecha y lugar:

1. Objetivos

**Objetivo(s) Investigación**
**Evaluar el efecto de las intervenciones digitales basadas en cápsulas audiovisuales educativas cuyo fin es la entrega de información necesaria para la prevención de infecciones de transmisión sexual/VIH.**
**Objetivo(s) Grupo Focal - ESTUDIANTES**
**Recoger y registrar información de estudiantes sobre experiencias y percepciones en torno a sexualidad, riesgo y campañas de prevención de ITS/VIH**

2. Identificación del moderador

**Moderador**
**Eduardo**

Observador
Giuliano

3. Participantes

Lista de asistentes Grupo focal	
1.	
2.	
3.	
4.	
5.	
6.	
7.	
8.	
9.	
10.	

4. Preguntas guías

Preguntas guías	
**1.**	**¿Qué es para Ustedes la sexualidad?**<br>**¿Es lo mismo: sexo, sexualidad, erotismo?**<br>**¿Quién les guío en el proceso de acercamiento a estos temas?**<br>**¿Qué rol tuvo: la familia, la iglesia, la escuela?**
**2.**	**¿Tuvieron educación sexual en sus colegios?**<br>**¿Lo consideran necesario?**<br>**¿Cómo fueron dichas experiencias educativas?**<br>**¿Mejoras? ¿Modificaciones?**

**3.**	**¿Saben lo que es una Infección de transmisión sexual?**<br><br>**¿Qué conocen del VIH y del Sida?**<br><br>**¿Conocen métodos de prevención de infecciones de transmisión sexual?**<br><br>**¿Cómo se informan de ellos?**<br><br>**¿Cómo acceden a preservativos, exámenes, consultorías?**<br><br>**¿Conocen las condoneras de la USACH?**<br><br>**¿Conocen el centro de salud de la USACH?**
**5**	**¿Cómo definirían el riesgo?**<br><br>**¿Cuál es su percepción acerca de los riesgos asociados a las ITS y VIH?**
**6.**	**¿Recuerdan alguna campaña de prevención ITS o de VIH/SIDA?**<br><br>**¿Qué opinan de las campañas de prevención de ITS o de VIH/SIDA?**<br><br>**¿Cómo debiera ser una campaña de promoción en salud sexual y prevención en ITS y VIH/SIDA? ¿Qué debiera decir?**<br><br>**¿Quiénes debieran ser sus protagonistas?**<br><br>**¿Por cuáles plataformas difundirla? Utilización de RRSS, plataformas digitales y videos.**<br><br>**¿Qué incentivos existen para el uso de preservativos y toma de exámenes preventivos en ITS/VIH?**

5. Pauta de chequeo

Chequear	Si/No
Lugar adecuado en tamaño y acústica	
Asistentes sentados en U en la sala	
Escarapelas con identificación de asistentes	
Refrigerios adecuados; no interrumpen el desarrollo de la actividad	
Registro de la información (grabadora y/o cámara de video)	
Reunión entre 60 y 120 minutos	
Explicita en un comienzo objetivos y metodología de la reunión a participantes	
Moderador escucha y utiliza la información que está siendo entregada. Permite que todos participen	
Moderador respeta tiempo para que los participantes desarrollen cada tema	
Se cumplen los objetivos planteados para esta reunión	

GUIDE FOR FOCUS GROUPS

Focus Group No .:

Date and place:

1. Objectives

Objective(s) Research
To evaluate the effect of digital interventions based on educational audiovisual capsules whose purpose is the delivery of information necessary for the prevention of sexually transmitted infections/HIV.
**Objective(s) Focus Group - STUDENTS**
Collect and record information from students about experiences and perceptions regarding sexuality, risk and STI/HIV prevention campaigns

2. Moderator ID

Moderator
Eduardo

Observer
Giuliano

3. Participants

List of attendees Focus group	
1.	
2.	
3.	
4.	
5.	
6.	
7.	
8.	
9.	
10.	

4. Guiding questions

Guiding questions	
**1.**	**What is sexuality for you?**<br><br>**Is it the same: sex, sexuality, eroticism?**<br><br>**Who guided you in the process of approaching these issues?**<br><br>**What role did it have: the family, the church, the school?**
**2.**	**Did they have sex education in their schools?**<br><br>**Do you consider it necessary?**<br><br>**How were these educational experiences?**<br><br>**Improvements? Modifications?**
**3.**	**Do you know what a sexually transmitted infection is?**<br><br>**What do you know about HIV and AIDS?**<br><br>**Do they know methods of prevention of sexually transmitted infections?**<br><br>**How are they informed?**<br><br>**How do they access condoms, exams, consultations?**<br><br>**Do you know the USACH condoms ?**<br><br>**Do you know the USACH health center?**

**5**	**How would you define risk?**<br><br>**What is your perception about the risks associated with STIs and HIV?**
**6.**	**Do you remember any STI or HIV/AIDS prevention campaign?**<br><br>**What do you think of STI or HIV/AIDS prevention campaigns?**<br><br>**How should a sexual health promotion and STI and HIV/AIDS prevention campaign be?**<br><br>**What should it say?**<br><br>**Who should be its protagonists?**<br><br>**On which platforms to spread it? Use of RRSS, digital platforms and videos.**<br><br>**What incentives exist for the use of condoms and taking preventive tests for STIs/HIV?**

5. Check guideline

**check**	**Ok**
**Suitable place in size and acoustics**	
**Attendees sitting in a U in the room**	
**Badges with identification of attendees**	
**Adequate snacks; do not interrupt the development of the activity**	
**Recording of information (recorder and/or video camera)**	

**Meeting between 60 and 120 minutes**	
**Explain the objectives and methodology of the meeting to the participants at the beginning**	
**Moderator listens and uses the information that is being delivered. Let everyone participate**	
**Moderator respects time for participants to develop each topic**	
**The objectives set for this meeting are met**	
