# Supplementary material for: Barriers and facilitators for safe sex behaviors in students from universidad de Santiago de Chile (USACH) through the COM-B model
Source: BMC Public Health. 2023 Apr 11;23:677. doi: 10.1186/s12889-023-15489-y (PMC10088188; doi:10.1186/s12889-023-15489-y)
Supplement: Supplementary file 2 — Supplementary Material 2 [file 12889_2023_15489_MOESM2_ESM.pdf]

**ANNEX 1**			
**Consolidated criteria for reporting qualitative studies (COREQ): 32-item checklist**			
**No**	**Item**	**Guide questions/description**	**COREQ CRITERIA IN Barriers and facilitators for safe sex behaviors in students from Universidad de Santiago de Chile (USACH) through the COM-B Model**
**Domain 1: Research team and reflexivity**			
Personal Characteristics			
1.	Interviewer/facilitator	Which author/s conducted the interview or focus group?	Eduardo Leiva Pinto
2.	Credentials	What were the researcher's credentials? E.g. PhD, MD	Anthropologist, Master Political Philosophy, PhD (e) Social Justice
3.	Occupation	What was their occupation at the time of the study?	University professor
4.	Gender	Was the researcher male or female?	Male
5.	Experience and training	What experience or training did the researcher have?	The researcher has training in qualitative, quantitative and mixed methods. He has conducted qualitative health research.
Relationship with participants			
6.	Relationship established	Was a relationship established before the start of the study?	The researchers did not know the participants in the focus groups. To facilitate rapprochement with the study participants, it was decided to do so with the help of the University's Gender and Sexuality Student Society (VGS for its acronym in Spanish). The VGS collaborated in the convening and selection of the students participating in the focus groups.
7.	Participant knowledge of the interviewer	What did the participants know about the researcher? e.g. personal goals, reasons for doing the research	The participants did not know the interviewer/facilitator. The facilitator/interviewer explains at the beginning the objectives and methodology of the meeting to the participants. The interviewer/facilitator reported general aspects of the study: title, investigators, financing. In addition, it was emphasized that there were no right or wrong answers, and that the important thing was their experiences and opinions on the topics discussed.
8.	Interviewer characteristics	What characteristics were reported about the interviewer/facilitator? e.g. Bias, assumptions, reasons and interests in the research topic	Before starting each focus group, the interviewer/facilitator read the 'Informed Consent' (validated by the Institutional Ethics Committee of the University of Santiago de Chile), commented on the confidentiality of the data and answered questions.
**Domain 2: study design**			
Theoretical framework			

9.	Methodological orientation and Theory	What methodological orientation was stated to underpin the study? e.g. grounded theory, discourse analysis, ethnography, phenomenology, content analysis	Content analysis
Participant selection			
10.	Sampling	How were participants selected? e.g. purposive, convenience, consecutive, snowball	The study used a purposive sample
11.	Method of approach	How were participants approached? e.g. face-to-face, telephone, mail, email	To facilitate rapprochement with the study participants, it was decided to do so with the help of the University's Gender and Sexuality Student Society (VGS for its acronym in Spanish). VGS invited the students to participate in the focus groups. For this, the VGS used their emails and social networks.
12.	Sample size	How many participants were in the study?	20
13.	Non-participation	How many people refused to participate or dropped out? Reasons?	8 students did not show up - 12 students refused to participate
Setting			
14.	Setting of data collection	Where was the data collected? e.g. home, clinic, workplace	The information was collected in the work space of the VGS.
15.	Presence of non-participants	Was anyone else present besides the participants and researchers?	Participants: Eduardo Leiva Pinto, facilitator/interviewer; Giuliano Duarte Anselmi, principal investigator, observer
16.	Description of sample	What are the important characteristics of the sample? e.g. demographic data, date	Sociodemographic data were collected for sample characterization: age, sex, gender, studies.
Data collection			
17.	Interview guide	Were questions, prompts, guides provided by the authors? Was it pilot tested?	Guides were used
18.	Repeat interviews	Were repeat interviews carried out? If yes, how many?	No
19.	Audio/visual recording	Did the research use audio or visual recording to collect the data?	Audio recording was used
20.	Field notes	Were field notes made during and/or after the interview or focus group?	Yes, field notes were taken during and after the focus groups.
21.	Duration	What was the duration of the interviews or focus group?	2 hours each
22.	Data saturation	Was data saturation discussed?	It was discussed and it was decided to balance the value of information delivered with its saturation.

23.	Transcripts returned	Were transcripts returned to participants for comment and/or correction?	No. The focus group discussions were audio recorded. The recordings were then transcribed, word for word, by a professional hired to do the job.<br>The transcript was later reviewed by Eduardo Leiva Pinto, listening to the recording again.<br>The recordings of the focus groups will be kept and guarded by the principal investigator and co-investigator
**Domain 3: analysis and findings**			
Data analysis			
24.	Number of data coders	How many data coders coded the data?	Three (FV, OF, VZ)
25.	Description of the coding tree	Did authors provide a description of the coding tree?	Yes
26.	Derivation of themes	Were themes identified in advance or derived from the data?	Analysts identified "barriers" (situations where safer sex behavior was not possible) and "facilitators" (situations where safer sex behavior was easier), which they identified independently and blindly from the transcript.
27.	Software	What software, if applicable, was used to manage the data?	No software was used to content analysis
28.	Participant checking	Did participants provide feedback on the findings?	No
Reporting			
29.	Quotations presented	Were participant quotations presented to illustrate the themes / findings? Was each quotation identified? e.g. participant number	Yes, citations were filed; they were codified, keeping anonymity.
30.	Data and findings consistent	Was there consistency between the data presented and the findings?	There was consistency
31.	Clarity of major themes	Were major themes clearly presented in the findings?	Yes in discussion we describe with clarity the relevance of our findings related with major themes of COM-B models
32.	Clarity of minor themes	Is there a description of diverse cases or discussion of minor themes?	-
